# Touraine-Solente-Gole syndrome: pathogenic variant in SLCO2A1 presented with polyarthralgia and digital clubbing

**DOI:** 10.1186/s12969-023-00831-w

**Published:** 2023-05-24

**Authors:** Rafaela Nicolau, Tiago Beirão, Francisca Guimarães, Francisca Aguiar, Sara Ganhão, Mariana Rodrigues, Ana Grangeia, Iva Brito

**Affiliations:** 1 Rheumatology department, Centro Hospitalar Tondela-Viseu, Viseu, +351915161576 Portugal; 2 Faculty of Medicine, University of Porto, Porto, Portugal; 3 Rheumatology department, Centro Hospitalar Vila Nova de Gaia/Espinho, Porto, Portugal; 4 Pediatric department, Centro Hospitalar Entre Douro e Vouga, Santa Maria da Feira, Portugal; 5 Pediatric and young adult Rheumatology unit, Centro Hospitalar Universitário de São João, Porto, Portugal; 6 Human Genetics Department, Centro Hospitalar Universitário São João, Porto, Portugal

**Keywords:** Primary hypertrophic osteoarthropathy, Touraine Solente Gole syndrome, Adolescent, SLCO2A1 gene

## Abstract

**Background:**

Primary Hypertrophic Osteoarthropathy (PHO), also known as Touraine-Solente-Gole Syndrome, is a rare, multisystemic autosomal recessive disorder caused by pathogenic variants in the 15-hydroxyprostaglandin dehydrogenase *(HPGD)* or Solute Carrier Organic Anion Transporter Family Member 2A1 (*SLCO2A1)* genes. However, autosomal dominant transmission has also been described in some families with incomplete penetrance. PHO usually starts in childhood or adolescence, presenting with digital clubbing, osteoarthropathy, and pachydermia. We described a complete form of the syndrome in a male patient with a homozygous variant in the SLCO2A1 gene (c.1259G > T).

**Case presentation:**

A 20-year-old male was referred to our Pediatric Rheumatology Clinic with a five-year history of painful and swollen hands, knees, ankles and feet, prolonged morning stiffness and relief with non-steroidal antiinflammatory drugs. He also reported late onset facial acne and palmoplantar hyperhidrosis. Family history was irrelevant and parents were non-consanguineous. On clinical examination, he presented clubbing of the fingers and toes, moderate acne and marked facial skin thickening with prominent scalp folds. He had hand, knee, ankles and feet swelling. Laboratory investigations showed elevated inflammatory markers. Complete blood count, renal and hepatic function, bone biochemistry were normal, as well as immunological panel. Plain radiographs revealed soft tissue swelling, periosteal ossification and cortical thickening of the skull, phalanges, femur and toe acroosteolysis. Due to the absence of other clinical signs suggesting a secondary cause, we suspected PHO. A genetic study revealed a likely pathogenic variant, c.1259G > T(p.Cys420Phe), in homozygosity in the *SLCO2A1* gene, thus confirming the diagnosis. The patient started oral naproxen with significant clinical improvement.

**Conclusions:**

PHO should be kept in the differential diagnosis of inflammatory arthritis affecting children, often misdiagnosed as Juvenile Idiopathic Arthritis (JIA). To the best of our knowledge, this is the second genetically confirmed case of PHO in a Portuguese patient (first variant c.644 C > T), both made at our department.

## Background

Primary Hypertrophic Osteoarthropathy (PHO), also known as Touraine-Solente-Gole Syndrome and pachydermoperiostosis, is a very rare congenital disease and accounts 3–5% of all cases of hypertrophic osteoarthropathy [1]. Three major clinical manifestations have been described: digital clubbing, periostosis and skin thickening (pachydermia). A variety of associated clinical manifestations were reported, including polyarthritis, *cutis verticis gyrata*, seborrhea, hyperhidrosis and myelofibrosis. It was initially described in 1935 by Touraine, Solente and Golé, who recognized 3 forms of presentation: a complete form with pachydermia and periostitis, an incomplete form with evidence of bone abnormalities but lacking pachydermia, and the fruste form with prominent pachydermia and minimal-to-absent skeletal changes. Its diagnosis can be made only after careful exclusion of secondary causes, such as underlying cardiopulmonary diseases and malignancies [2, 3].

PHO typically affects males, with a male to female ratio of 9:1. It begins during childhood or adolescence and may stabilize after 5–20 years, or progress gradually. Cases have been reported with an autosomal recessive inheritance pattern with pathogenic variants in HPGD gene, which encodes a prostaglandin E2 (PGE2) catabolizing enzyme, and in SLCO2A1 gene, which encodes prostaglandin transporter (PGT) responsible for uptake of PGE2. However, autosomal dominant transmission has also been described in some families with incomplete penetrance [4].

## Case presentation

We report a case of an otherwise healthy 20-year-old caucasian male patient, born to non-consanguineous healthy Portuguese parents. He was referred to our Pediatric and Young Adult Rheumatology Clinic for suspected inflammatory artropathy, due to a five-year history of painful and swollen hands, knees, ankles and feet. The pain had an inflammatory pattern, with prolonged morning stiffness and prompt relief with non-steroidal antiinflammatory drugs. He also reported late onset facial acne and palmoplantar hyperhidrosis. There were no constitutional symptoms, rashes, ulcers, ocular, gastrointestinal, cardiac or respiratory complaints.

**Fig. 1:**
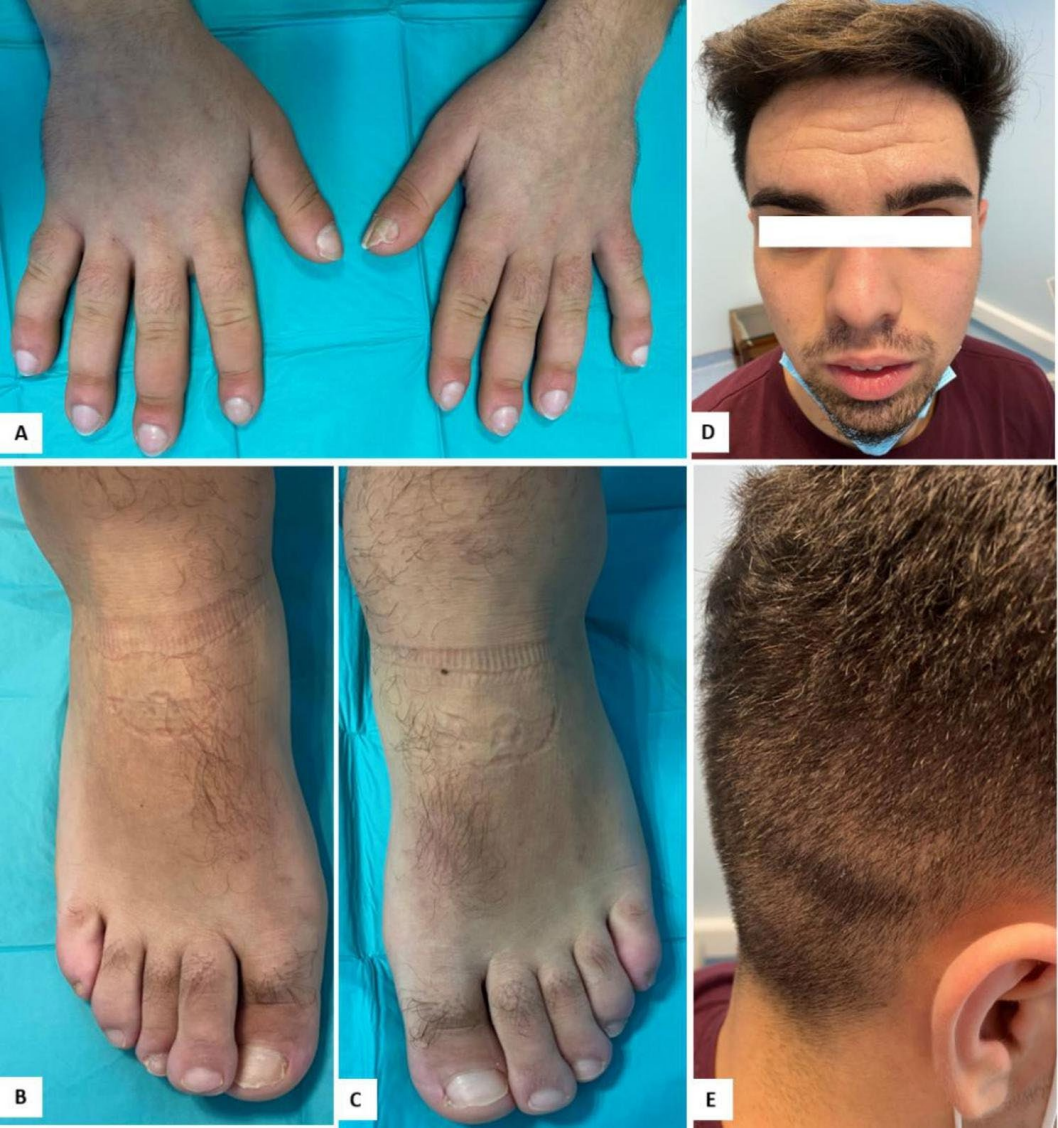
Digital clubbing of the hands (**A**) and toes (**B**) with diffuse edema of ankles. (**C**) Coarse facial features, thickening of the skin and prominent skin folds on the forehead and scalp (**D**)

**Fig. 2:**
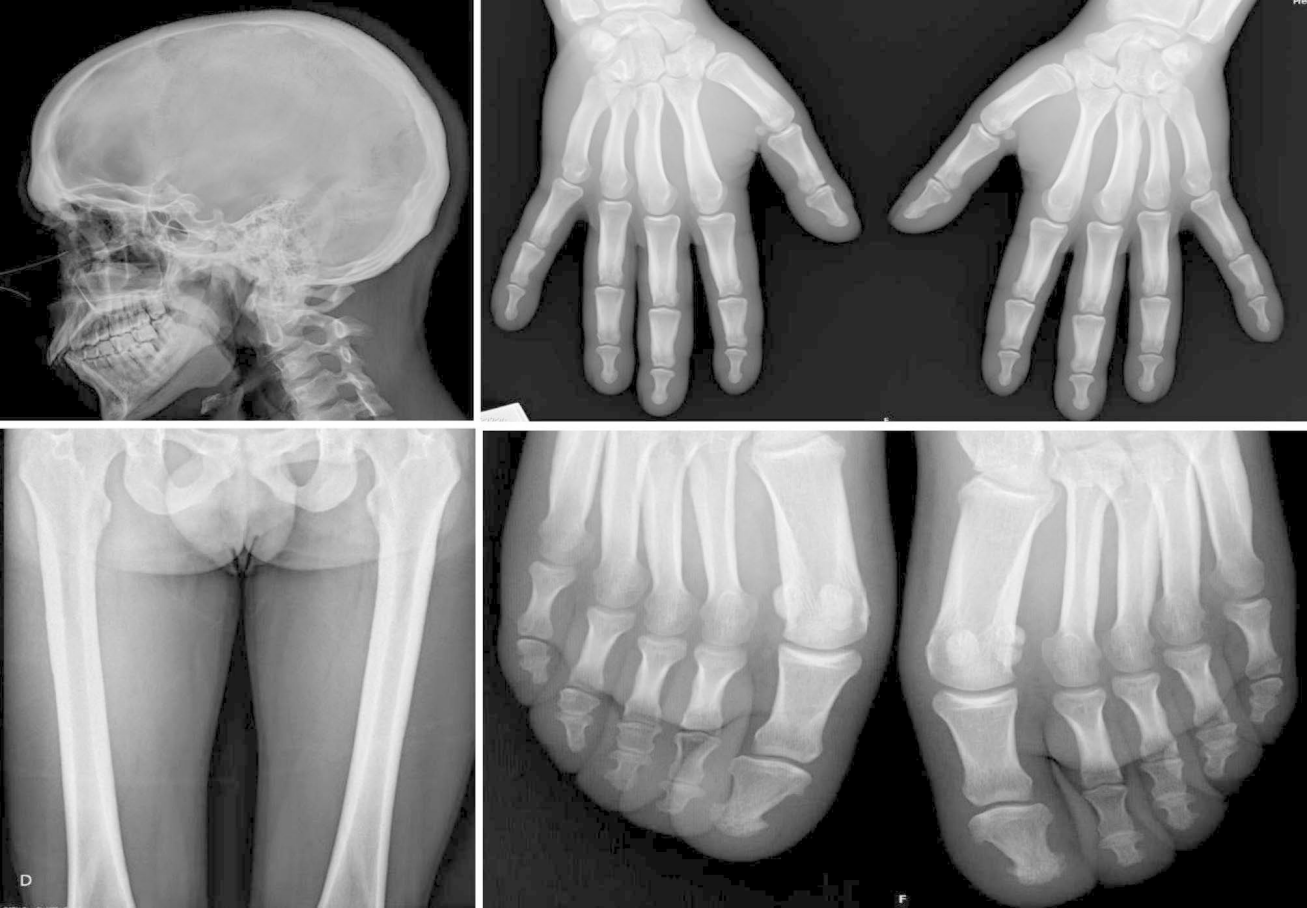
Plain radiographs show marked soft tissue swelling and periosteal hypertrophy with subperiosteal bone formation of the skull, femur and phalanges

On clinical examination, he presented painless clubbing of the fingers and toes, moderate acne and marked facial skin thickening with prominent forehead and scalp folds. He had hand, knee, ankles and feet soft swelling (Fig. 1). Plain radiographs revealed soft tissue swelling, periosteal ossification and cortical thickening of the skull, phalanges, femur and toe acroosteolysis (Fig. 2). Ultrasound showed no knee or ankle joint effusions. There was a layer of irregular echogenic tissue surrounding the femur and tibia, without Doppler signal.

Routine laboratory tests showed increased levels of C-reactive protein (CRP) (35.5 mg/L, normal range < 3 mg/L) and erythrocyte sedimentation rate (ESR) (27 mm/h, normal range 0–15 mm/h). Complete blood count, renal and hepatic function, bone biochemistry were normal, as well as immunological panel. Normal thyroid function tests, growth hormone, and insulin-like growth factor ruled out endocrinopathy. An abdominal ultrasound, electrocardiogram, echocardiogram, plain chest film, and tuberculin skin test were also performed to exclude secondary causes, which were unremarkable. Plasma PGE levels may be useful in suspected PHO cases, however, this was not available in our centre.

Clinical and imagiological findings were compatible with a diagnosis of hypertrophic osteoarthropathy, so molecular analysis of HPGD and SLCO2A1 genes was required. After obtaining a written consent, peripheral blood samples were obtained. Genomic DNA was extracted from peripheral blood leukocytes using standard techniques. The analysis performed was PCR amplification and sequencing of all coding exons, and flanking intronic regions, of the HPGD and SLCO2A1 genes. No HPGD mutation was found, while SLCO2A1 gene analysis detected c.1259G > T mutation in a homozygous state, in exon 9 of the gene, which causes the exchange of the highly conserved amino acid cysteine by a phenylalanine in 420 position in the protein. PHO type 2 diagnosis was made.

Therapy with naproxen 500 mg/day was started and is currently maintained, with significant clinical improvement. ESR and CPR subsequently declined (19 mm/h and 21 mg/L, respectively).

## Discussion

To the best of our knowledge, this is the second genetically confirmed case of PHO in a Portuguese patient (first variant c.644 C > T), both made at our department. This homozygote variant of SLCO2A1 gene has been previously described by Diggle et al. in 4 brothers with PHO from a consanguineous hispanic family of colombian ancestry [4, 5].

As highlighted in previous literature, PHO is categorized into two different types: PHO type 1 and 2, caused by pathogenic variants in HPGD and SCLCO2A1 genes, respectively. Both genes deficiency causes increased level of PGE2, due to a defect in degradation (HPGD) and transport (SCLCO2A1) of PGE2. High concentrations of plasma PGE2 promote osteoblast activity, fibroblast growth and increased collagen and extracellular matrix production, which is the major characteristic of bone and soft tissue findings. PHO type 1 patients usually present digital clubbing in early childhood, and other symptoms, such as patent ductus arteriosus or delayed closure of the cranial sutures. PHO type 2 individuals are diagnosed after puberty or in early adulthood. Pachydermia occurs in both types, but severe cutis gyrata only in the SLCO2A-deficient group. Radiologically, periosteal bone deposition is seen in both groups, but acroosteolysis is much more prominent in the HPGD-deficient group. Myelofibrosis has been associated to SLCO2A1 group [6, 7].

Our patient presented a complete form of hypertrophic osteoarthropathy with onset in puberty, as seen in patients with homozygous SCLO2A1 mutations. Clinical history, physical examination, and plain radiographs were very suggestive. Plain films permit the evaluation of periostosis, with radius, ulna, tibia and fibula most commonly affected. Another frequent finding is acro-osteolysis of the distal phalanges.

The differential diagnosis depends on the presenting phenotype and age of onset and includes secondary hypertrophic osteoarthropathy, thyroid acropachy, and acromegaly. Secondary causes of hypertrophic osteoarthropathy, which are much more frequent (95% cases), especially those associated to lung neoplasia and, to a lesser degree, to hepatic cirrhosis, cardiopathy and chronic obstructive pulmonary disease, should be excluded, especially when dermatological signs are not prominent. The presence of arthralgia or arthritis might suggest systemic connective tissue diseases, such as JIA. In this case, osteoarticular ultrasound showed no joint effusions, but instead a layer of irregular echogenic tissue in thickness surrounding the femur and tibia without increased vascularity on Doppler. Awareness of the significance of digital clubbing under these circumstances and absence of synovitis on ultrasound is likely to prevent misdiagnosis.

After excluding secondary causes of hypertrophic osteoarthropathy and other differential diagnoses, genetic studies may be useful to confirm the diagnosis and classify the PHO type. The identification of the mutation contributes not only to confirm diagnosis, but also to support future genetic counseling and prevention of associated disease complications.

Currently available therapeutic options are palliative and directed toward amelioration of the patient complaints. Clinical improvement of musculoskeletal symptoms can be achieved by nonsteroidal anti-inflammatory drugs, as verified in our patient. PGE2 is a lipid mediator derived from arachidonic acid through the action of enzymes, including the ubiquitous tissue constitutive isoform cyclooxigenase (COX-1) and the inflammatory/tumor-induced isoform (COX-2). Inflammatory marker levels including CRP and ESR were found to be high at baseline and subsequently declined after COX-2 inhibitor treatment, signifying a decline in inflammation with decreasing levels of PGE2. In addition, bisphosphonates, hydroxychloroquine, tamoxifen citrate, octreotide and colchicine have been reported as effective therapies in refractory cases. Plastic surgery should be reserved for extreme cases to remove excess of facial skin or to reduce finger clubbing [8, 9].

Finally, it needs to be underlined that a patient presenting with clubbing should be classified as having PHO only after careful scrutiny fails to reveal an underlying secondary cause. Meticulous follow up is mandatory to screen for rare complications like myelofibrosis.

## Conclusion

The report in question emphasis the importance of considering PHO as a possible diagnosis in Pediatric and Young Adult Rheumatology, due to prognostic and therapeutic implications.

## Data Availability

The datasets generated during the current study are available from the corresponding author on reasonable request.

## Abbreviations

PHO: Primary Hypertrophic Osteoarthropathy
HPGD: Hydroxyl-Prostaglandin Dehydrogenase
SLCOA21: Solute Carrier Organic Anion Transporter Family Member 2A1
PGE2: Prostaglandin E2
PGT: Prostaglandin transporter
JIA: Juvenile Idiopathic Arthritis
COX: Cyclooxygenase
