# Correction to: Touraine-Solente-Gole syndrome: pathogenic variant in SLCO2A1 presented with polyarthralgia and digital clubbing

**DOI:** 10.1186/s12969-023-00850-7

**Published:** 2023-06-26

**Authors:** Rafaela Nicolau, Tiago Beirão, Francisca Guimarães, Francisca Aguiar, Sara Ganhão, Mariana Rodrigues, Ana Grangeia, Iva Brito

**Affiliations:** 1 Rheumatology department, Centro Hospitalar Tondela-Viseu, Viseu, +351915161576 Portugal; 2 Faculty of Medicine, University of Porto, Porto, Portugal; 3 Rheumatology department, Centro Hospitalar Vila Nova de Gaia/Espinho, Porto, Portugal; 4 Pediatric department, Centro Hospitalar Entre Douro e Vouga, Santa Maria da Feira, Portugal; 5 Pediatric and young adult Rheumatology unit, Centro Hospitalar Universitário de São João, Porto, Portugal; 6 Human Genetics Department, Centro Hospitalar Universitário São João, Porto, Portugal

**Correction to: Pediatric Rheumatology (2023) 21:48**

10.1186/s12969-023-00831-w

The consent for publication section in the original publication was incorrect. The incorrect and correct information is listed in this correction article. The original article has been updated.

Incorrect

Not applicable.

Correct

Publication consent was obtained from all participants.

